# Study on the safety and effectiveness of low-dose vs. regular-dose fondaparinux in preventing venous thromboembolism prophylaxis following total knee arthroplasty

**DOI:** 10.3389/fcvm.2023.1195322

**Published:** 2023-07-06

**Authors:** Ping-bo Chen, Jing Wang, Lei Wang, Shou-liang Xiong, Chao Wang, Xin Yang, Cong-ming Li, Qiang Wang, Yin-chang Zhang

**Affiliations:** ^1^ Department of Orthopaedic Surgery, The First Affiliated Hospital of Wannan Medical College, Wuhu, China; ^2^ Department of Gastroenterology, The First Affiliated Hospital of Wannan Medical College, Wuhu, China

**Keywords:** fondaparinux, venous thromboembolism, total knee arthroplasty, wound complication, blood loss, blood transfusion, deep venous thrombosis

## Abstract

**Background:**

This study aims to evaluate the effectiveness and safety of low-dose (1.5 mg) fondaparinux for venous thromboembolism (VTE) prophylaxis in patients post-total knee arthroplasty (TKA).

**Methods:**

We retrospectively identified 314 patients who carried out the primary TKAs and received fondaparinux for VTE chemoprophylaxis between July 2020 and December 2021. A total of 141 TKA patients were excluded according to the exclusion criteria. Two groups of patients were established: the low-dose group included 84 patients who injected 1.5 mg of fondaparinux, and the regular-dose group included 89 patients who injected 2.5 mg of fondaparinux. The pre-operative blood analysis and coagulation assays were performed. The surgical time, the incidence of symptomatic VET, blood loss, wound complication, bleeding, drainage, and mortality of patients were determined and assessed.

**Results:**

The pre-operative blood analysis, body mass index, sex, age, and coagulation assays of patients in both groups were comparable. In terms of symptomatic pulmonary embolism and deep vein thrombosis, there was no significant difference (variation) between the two groups. However, patients in both groups showed a substantial difference in terms of blood loss, drain volume, wound complication, and transfusion rate.

**Conclusion:**

In prevention of VET in patients post-TKA, low-dose fondaparin is as effective as conventional dose fondaparinux. A significant decrease in blood loss, post-surgical transfusion rates, and wound complications were detected in patients given low-dose fondaparinux compared to those receiving regular-dose fondaparinux.

## 1. Introduction

Patients suffering from degenerative joint conditions have benefited greatly from total knee arthroplasty (TKA) in the term of quality of life. Nevertheless, the patients post-TKA are predisposed to venous thromboembolism (VTE) events (1). VTE is an uncommon but possibly fatal complication of TKA (2). The advent of modern chemoprophylaxis has reduced post-TKA VTE incidence. Nevertheless, 0.5%–1.0% of post-TKA patients will develop VTE (3).

Fondaparinux, an anticoagulant drug, is a synthetic pentasaccharide which can block factor Xa activity selectively, compared with low-molecular-weight (LMWHs) is not selective, acting on several different factors in the coagulation cascade (4). It's interesting to note that fondaparinux, a selective indirect antithrombin-dependent factor Xa inhibitor, has been demonstrated to have a larger net therapeutic effect than LMWH in the prevention of VTE. Moreover, the use of fondaparinux in clinical practice is increasing due to a more feasible dosing regimen compared to LMWH (5). It is now well accepted that Fondaparinux is both safe and efficacious for preventing VTE in patients after undergone a total joint replacement. Patients using Fondaparinux experience significantly decreased rates of VTE and heparin-induced thrombocytopenia (HIT) compared to those receiving LMWH (2).

Although fondaparinux's effectiveness has been thoroughly investigated, some documented adverse effects linked to VTE prophylaxis include bleeding and wound problems (6). In addition, a study has shown that the toxicity of fondaparinux is dose-dependent (5). The lowest effective dose of fondaparinux could be used to reduce bleeding and wound complications. However, if low-dose fondaparinux is effective as regular-dose fondaparinux in preventing VTE in patients post-TKA is still unclear. The American Association of Orthopedic Surgery guidelines recommends using 2.5 mg fondaparinux once a day to prevent VTE (7). However, it is yet uncertain low-dose fondaparinux is as effective as regular-dose for preventing VTE in patients who have undergone TKA in China.

Therefore, this research sought to compare the effectiveness and safety between two different dosages of fondaparinux in patients post-TKA.

## 2. Material and methods

### 2.1. Patients source

We retrospectively identified 314 patients who underwent elective primary TKA between July 2020 and December 2021, and received fondaparinux alone for VTE prophylaxis after TKA. During the study period, fondaparinux was prescribed as monotherapy for VTE prophylaxis after TKA.

**Figure 1:**
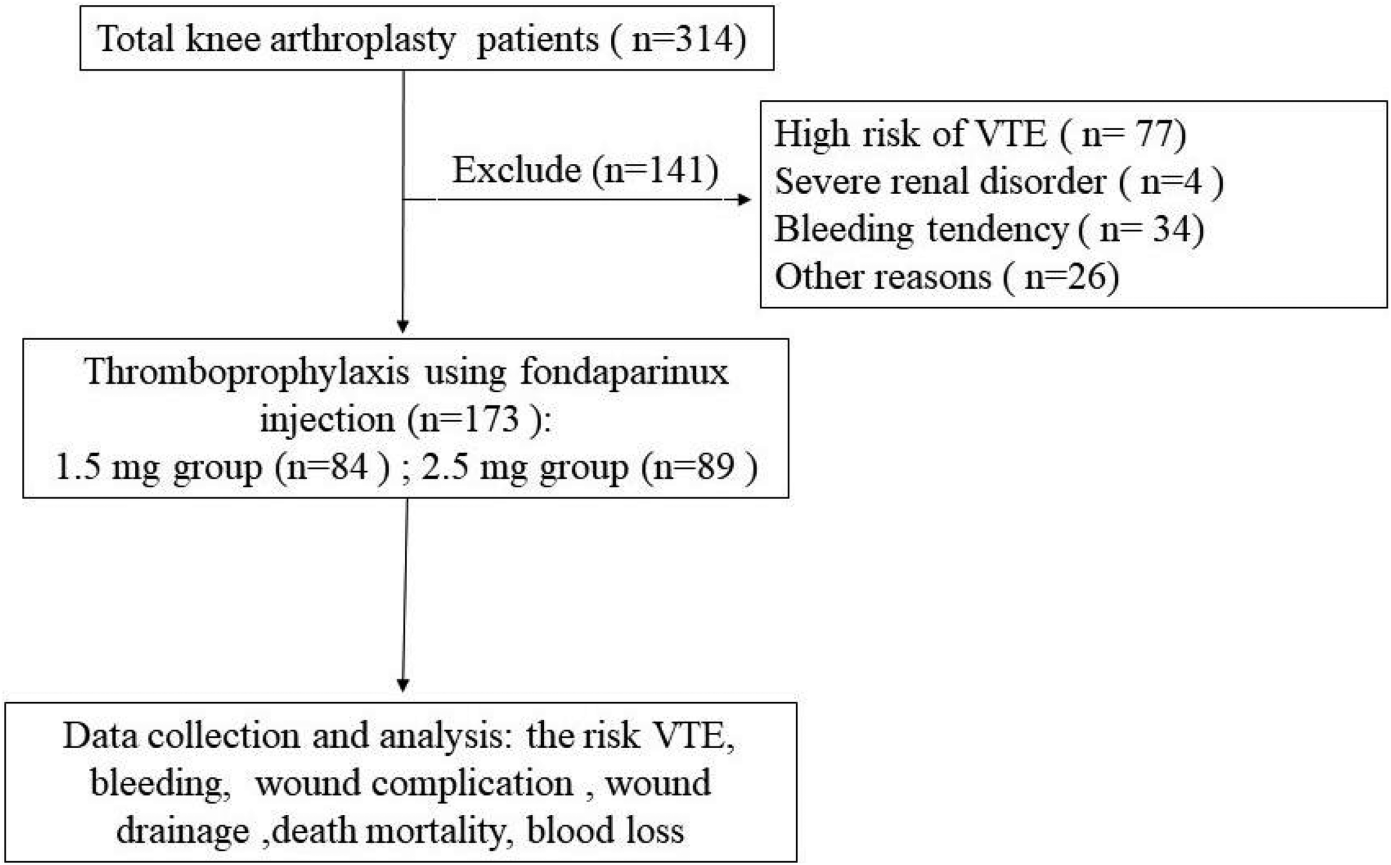
Patient selection and study design.

The exclusion criteria of the patients were as follows: (1) patients with high risk for VTE determined by the specialist according to the institution's guidelines, wherein patients having records of venous VTE, active malignancy, a confirmed prothrombotic disease, or a previous condition requiring anticoagulation were classified as high-risk patients. (2) Severe renal disorders (creatinine clearance <30 ml/min). (3) Bleeding tendency (abnormal platelet, partial thromboplastin time, or prothrombin time (PT); increased risk of hemorrhage, active gastric ulcer, or urinary tract bleeding in the previous year; hemorrhagic stroke, spinal, brain, or ophthalmologic surgical procedure in the previous six months). (4) Patients receiving other modalities of chemoprophylaxis with or without fondaparinux on the of the procedure. (5) Other factors like liver enzymes or bilirubin >3*normal; cancer in past 1 year, other than localized skin cancers. A total of 141 TKA patients were excluded (Figure 1).

Patients received fondaparinux once daily for 9 days after TKA and were grouped into two cohorts: a low-dose (1.5 mg) fondaparinux group (*n* = 84) and regular-dose (2.5 mg) fondaparinux group (*n* = 89).

### 2.2. Study setting

Patients were operated on under epidural or general anesthesia with an inflated tourniquet. A peripheral vein catheter with analgesic was inserted for 48 h post-surgery to control pain. One surgeon conducted the surgical procedures and was assisted by three other medical professionals. All patients received the cemented knee prosthesis (NexGen LPS, Zimmer, Warsaw, USA) of the same design. Following the insertion of a vacuum drain, the incision was stitched up in layers. The knee joint received an injection of 2 g of tranexamic acid mixed with 100 ml of ordinary saline, which was then suctioned out after 5 min. The tourniquet was released, and a tight compression bandage was placed over the wound. There was a 24 h retaining on the drain. Patients who required a catheter during surgery had their catheters removed on postoperative day 1.

The patient immediately began weight-bearing the next day after the surgery. Patients were asked to engage in a variety of physical activities, including knee flexion, isometric quadriceps, and ankle pumps. In addition, pneumatic compression stockings were given to all patients after surgery. Patients were administered fondaparinux subcutaneously (1.5 or 2.5 mg) daily after 6 h of surgery for 9 days (8, 9). All patients were treated with multimodal pain protocols like 40 mg parecoxib sodium was administered every 12 h and intravenous prophylactic antibiotics.

The routine blood analysis was performed on days 1, 3, and 7 post-surgery. It was determined that a transfusion was necessary when the patient's hemoglobin (Hb) content dropped below 70 g/L.

### 2.3. Data collection

Before beginning the surgery, we carried out standard blood tests such as measuring the patient's Hb content, platelet count and coagulation assays. Preoperatively, routine Duplex ultrasonography was conducted on each patient involving both lower limbs, and the classification of American Society of Anesthesiologists was collected.

The following were the study's primary outcomes: (1) The prevalence of VTE in patients taking fondaparinux (1.5 and 2.5 mg) within 90 days post-surgery. (2) Patients presenting pulmonary embolism (PE) and DVT symptoms, like calf pain, erythema, leg swelling, and tenderness, verified via a doppler ultrasonography for DVT. Computed tomography (CT) angiography or ventilation-perfusion were used to confirm PE.

The secondary outcomes of the study included the quantity of blood gathered in the drain × collectors within 24 h, post-surgical Hb levels, the decrease in Hb levels (pre-surgical Hb—72 h post-surgical Hb), and the number of blood transfusions in patients in both groups were compared.

The total amount of blood loss was calculated using the Hb balance technique (10):

BV = *K*_1_ × *H*^3^ + *K*_2_ × *W* + *K*_3_ (BV: blood volume in ml; Males: *K*_1_ = 0.3669, *K*_2_ = 0.03219, and *K*_3_ = 0.6041; Females: *k*_1 _= 0.3561, *k*_2 _= 0.03308, and *k*_3 _= 0.1833; *W* = Weight in kg; *H* = Height in m).

The loss volume of Hb = BV × (Hb_pre_ − Hb_post_) × 0.001 + Hbt (Hb_pre_: Hb value prior to surgery; Hb_post_: Hb value post-surgery on day 3; Hbt: total volume of blood transfusion)

$$\mathrm{Total}\;\mathrm{blood}\;\mathrm{loss}=1000\times \mathrm{total}\;\mathrm{Hb}\;\mathrm{loss}/Hb_{\mathrm{pre}}.$$

The tertiary outcomes of the study were as follows: The occurrence of wound complications in surgical patients receiving fondaparinux (1.5 or 2.5 mg) daily for VTE prevention, during 90 days postoperatively. Other hospital-related complications were also documented.

### 2.4. Statistical analysis

The SPSS version 23.0 was applied to all data analyses. Data were expressed as mean ± standard deviation. The two-tailed Student's *t*-test was employed for analyzing continuous variables assuming two samples with equal variance. To examine the relationships between categorical variables, Fisher's exact test was utilized. *P* < 0.05 denoted the significance level.

## 3. Results

**Figure 2:**
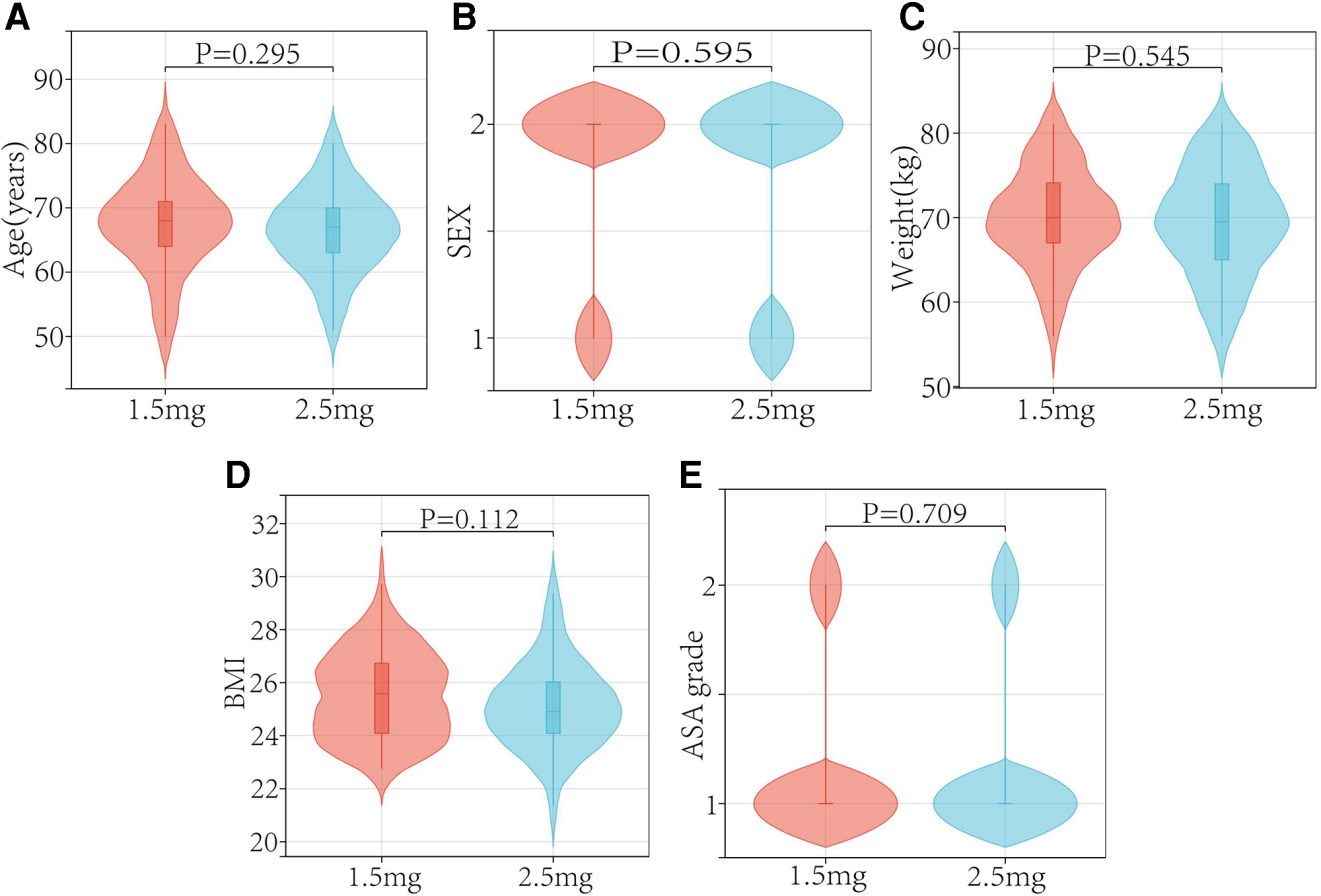
Patients demographic profiles in two group.

**Figure 3:**
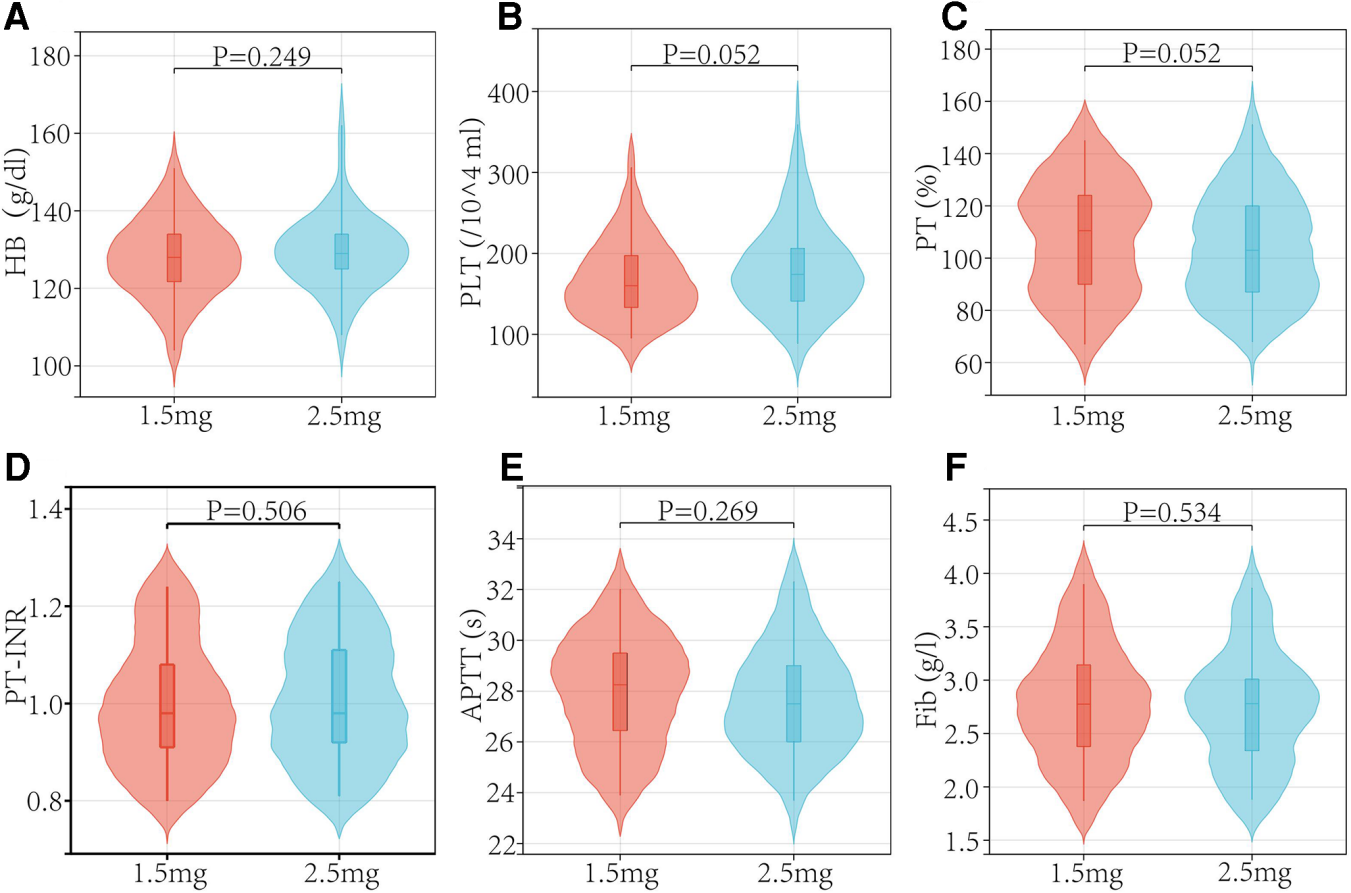
Preoperative blood analysis and coagulation assays.

In both the low and regular dose fondaparinux groups, patients had comparable demographics in respect of age, sex, weight, BMI, and ASA grade (Table 1 and Figure 2). There were not observed the significant difference (*P* > 0.05) in mean age, mean BMI, the gender distribution of patients, and the ASA grade in the two groups. No remarkable variation in the Hb level of patients between both groups was observed. In low and regular dose of fondaparinux groups, the mean pre-surgical HB value of patients was 127.85 g/dl and 129.49 g/dl, correspondingly (*P* = 0.200). No significant variation in the pre-surgical blood analysis (platelet count, Hb level), coagulation assays (%PT, PT-INR, APTT, fibrinogen), and time needed for the surgical procedure were observed in patients in both groups (Table 2 and Figure 3).

**Table 1:** Patients demographic profiles in two group.

Parameter	1.5 mg FPX group (*n* = 84) *n* (%) or mean ± SD (range)	2.5 mg FPX group (*n* = 89) *n* (%) or mean ± SD (range)	95% CI (lower to upper)		*P*-value
			1.5 mg	2.5 mg	
Age (years)	67.48 ± 7.07 (50–83)	66.40 ± 6.31 (51–80)	65.94–69.01	65.07–67.73	0.295
Sex (%)					0.595
Male	17 (20.2)	20 (22.5)			
Female	67 (79.7)	69 (77.5)			
Weight (kg)	70.07 ± 5.48 (56–81)	69.53 ± 5.19 (58–85)	68.89–71.26	68.23–70.84	0.545
BMI	25.52 ± 1.58 (22.77–29.74)	25.13 ± 1.54 (21.41–29.37)	25.17–25.86	24.81–25.46	0.112
ASA grade (%)					0.709
I	69 (82.1)	75 (84.3)			
II	15 (17.9)	14 (15.7)			

**Table 2:** Preoperative blood analysis and coagulation assays.

Parameter	1.5 mg FPX group (*n* = 84) *n* (%) or mean ± SD (range)	2.5 mg FPX group (*n* = 89) *n* (%) or mean ± SD (range)	95% CI (lower to upper)		*P*-value
			1.5 mg	2.5 mg	
HB (g/dl)	127.85 ± 9.68 (104–151)	129.49 ± 9.01 (108–162)	125.75–129.95	127.59–131.39	0.249
PLT (/10^4^ ml)	167.71 ± 56.65 (95–306)	182.71 ± 54.05 (89–359)	157.59–177.84	171.32–194.10	0.052
PT (%)	107.65 ± 21.32 (67–145)	104.03 ± 19.80 (68–151)	103.03–112.28	99.86–108.21	0.250
PT-INR	1.00 ± 0.12 (0.80–1.24)	1.01 ± 0.12 (0.81–1.25)	0.97–1.03	0.99–1.04	0.506
APTT (s)	28.00 ± 2.11 (23.9–32.0)	27.65 ± 21.02 (23.7–32.3)	27.54–28.45	27.22–28.07	0.269
Fib (g/L)	2.79 ± 0.53 (1.87–3.90)	2.74 ± 0.52 (1.88–3.86)	2.68–2.91	2.64–2.85	0.534

**Figure 4:**
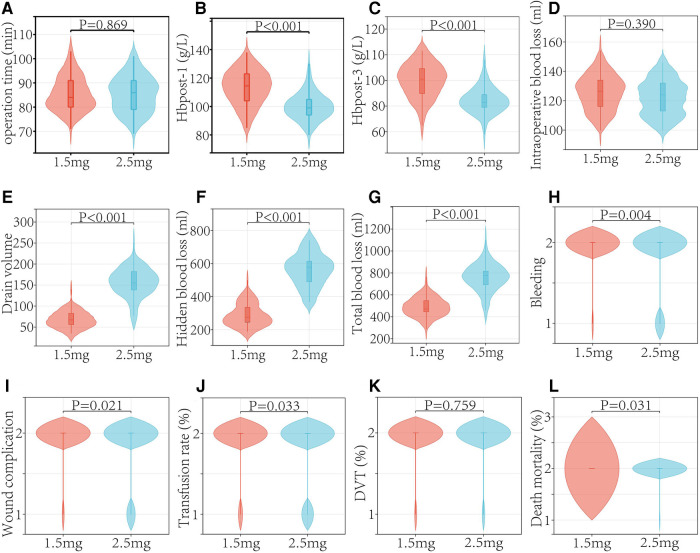
Analysis of outcomes in the two groups.

The findings showed that there was no significant difference in the incidence of DVT between the two groups (*P* = 0.795). There was no PE patient among the whole patient population (Table 3 and Figure 4).

**Table 3:** Analysis of outcomes in the two groups.

Parameter	1.5 mg FPX group (*n* = 84) *n* (%) or mean ± SD (range)	2.5 mg FPX group (*n* = 89) *n* (%) or mean ± SD (range)	95% CI (lower to upper)		*P*-value
			1.5 mg	2.5 mg	
Operation time (min)	85.55 ± 6.81 (71–103)	85.37 ± 7.25 (69–101)	84.07–87.02	83.84–86.90	0.869
Hb_post-1_ (g/L)	113.32 ± 11.86 (85–138)	100.11 ± 8.98 (80–130)	110.75–115.89	98.22–102.00	<0.001
Hb_post-3_ (g/L)	99.81 ± 12.84 (65–123)	84.60 ± 9.44 (58–116)	97.02–102.60	82.61–86.58	<0.001
Intraoperative blood loss (ml)	125.68 ± 11.47 (100–154)	122.00 ± 11.78 (100–146)	123.19–128.17	119.52–124.48	0.390
Drain volume (ml)	66.50 ± 19.17 (20–140)	156.94 ± 30.31 (77–250)	62.34–70.66	150.57–163.33	<0.001
Hidden blood loss (ml)	288.29 ± 66.77 (189–498)	561.20 ± 110.54 (368–739)	273.80–302.78	540.86–581.55	<0.001
Total blood loss (ml)	490.33 ± 80.97 (289–763)	758.74 ± 114.86 (459–1,100)	472.76–507.91	734.54–782.94	<0.001
Bleeding	2 (2.38%)	13 (16.61%)			0.004
Wound complication	3 (3.57%)	12 (13.48%)			0.021
Transfusion rate (%)	7.14% (6/84)	17.98% (17/89)			0.033
DVT (%)	3.57% (3/84)	4.49% (4/89)			0.759
PE (%)	0% (0/84)	0% (0/89)			N/A
Death mortality (%)	0% (0/84)	1.12% (1/89)			0.031

Patients had an 8.67% rate of bleeding. The fondaparinux groups (1.5 and 2.5 mg) had incidences of postoperative bleeding of 2.38% and 16.61%, respectively (*P* = 0.004). In 2.5 mg fondaparinux group, wound-related bleeding was observed in 10 patients, and GI bleeding was observed in 3 patients. Within 90 days after surgery, one patient in 2.5 mg fondaparinux group died from cerebral hemorrhage. Further, no mortalities were observed in patients who received 1.5 mg fondaparinux (*P* =** **0.031) (Table 3 and Figure 4).

There were 13 cases of early postoperative wound problems in the 2.5 mg fondaparinux group. Five patients appeared delayed healing or blistering; however, no patients needed re-admission or re-surgery. Two patients experienced a post-operative hematoma with extensive ecchymosis. Only one patient was re-admitted during the early postoperative period and was treated for erysipelas with intravenous antibiotics. Two patients in the group that received fondaparinux at a dosage of 2.5 mg had superficial infections that required two weeks of parenteral antibiotic treatment. Neither group of individuals had any severe infections (Table 3 and Figure 4).

The median intra-surgical blood loss values in patients receiving 2.5 and 1.5 mg fondaparinux were 122.00 and 125.68 ml, respectively (P =** **0.390). The mean total external drain output and wound drainage were significantly low in patients given fondaparinux at a dosage of 1.5 compared to those who received a dosage of 2.5 mg. Between the two groups, there were significant differences in the blood transfusion rates. Patients who received 2.5 mg of fondaparinux experienced a significantly greater total blood loss than those given 1.5 mg of fondaparinux. The numbers of patients requiring blood transfusion were 6 and 17 in the 1.5 mg and 2.5 fondaparinux groups correspondingly (Table 3 and Figure 4).

## 4. Discussion

Fondaparinux is well-studied and proven to be efficient and safe for VTE prevention after total joint arthroplasty (2). The American College of Chest Physicians Guidelines for the Prevention of DVT/PE recommends Fondaparinux as a Grade 1 B medication for preventing VTE in patients post-TKA (11). However, a previous study has shown severe complications like bleeding due to fondaparinux (12). Some studies have shown that the anticoagulant could cause bleeding and wound complications (13). Hence overdose and prolonged use of anticoagulants should be avoided (14). It is unknown, however, whether low-dosage or standard-dosage fondaparinux as the chemoprophylactic drug affects the VTE incidence in Chinese patients after TKA. Therefore, we sought to examine if the low-dose or regular-dose of fondaparinux as thromboprophylaxis for patients post-TKA affects the rate of post-surgical VTE, bleeding, wound complication, drainage, and mortality. Our results revealed significantly higher incidences of bleeding, wound complication, and wound drainage in patients receiving regular doses of fondaparinux. It's interesting to note that there was no discernible difference between these patients' later rates of VTE or mortality.

Our research has some advantages. Some strengths need to be considered when interpreting the results. All surgeries were performed at a single facility with standardized perioperative treatment, including anesthesia, prosthesis fixation, rehabilitation regimen, pain control, perioperative antibiotics, and other forms of care. Thus, potential confounders that alter patients' risk of developing VTE are minimized.

Our analysis shows low incidences of VTE within 90 days post-TKA. But we have only examined the incidences of symptoms related to VTE in patients, and we did not perform routine post-surgical screening for VTE in asymptomatic patients who underwent TKA since there is no impact on patient outcomes or clinical care as a result of this. Therefore, the occurrence of a thrombotic event in the absence of any symptoms may be undetected. Several reasons for the low incidence of VTE in our study include fondaparinux use, other multimodal protocols used for preventing VTE, like pneumatic compression during hospitalization, and early mobilization postoperatively. The VTE incidence from Japanese patients who had TKA and received personalized dosages of fondaparinux depending on their VTE risk was studied, and the results revealed that the VTE incidence was 34.2%, 21.3%, 16.2%, and 9.5% in patients receiving 0.75-, 1.5-, 2.5-, and 3.0 mg of fondaparinux, correspondingly (9).

A previous study has shown that prolonged wound drainage post-total joint arthroplasty significantly increases the risk of wound complications in patients (15). Prolonged wound drainage delays wound healing and blistering in patients. The Weiss method was used to calculate wound drainage; the surgical wound was dry in 83% of patients receiving aspirin and 77% of patients who received enoxaparin on day 5 post-surgery (15). Another research found that by day 5 after surgery, the wounds of 83% of patients treated with 2.5 mg of fondaparinux were dry (16). Our results revealed that the surgical wounds of 90% of patients who were given 1.5 mg fondaparinux were dry on day 5 post-surgery. Together, these data imply that the risk of wound drainage and consequent wound complications in patients given 1.5 mg of fondaparinux was comparable to those receiving 2.5 mg.

A study has reported that factor-Xa inhibitors increase the incidence of bleeding and wound complications after total joint replacement (6). Two clinical trials have reported significantly higher incidence rates of major and minor bleeding in patients treated with fondaparinux compared to LMWH (17). Based on our findings, patients who were given 1.5 mg of fondaparinux had significantly lower rates of bleeding and wound complications than those who were given 2.5 mg of fondaparinux. The number of complications experienced by patients who received 1.5 mg fondaparinux was much lower than those getting 2.5 mg fondaparinux, as shown by our findings. This complements existing findings and provides credence to the need for more research into complication and risk stratification to determine the optimal fondaparinux dose for VTE prevention post-TKA in Chinese patients.

## 5. Conclusion

In conclusion, fondaparinux could be used for thromboprophylaxis in patients post-TKA. Our findings illustrate that low-dose fondaparinux is not less effective as a preventive medication in patients after TKA compared to standard-dose fondaparinux. Additionally, patients administered 2.5 mg of fondaparinux daily may have increased occurrences of bleeding and wound complications relative to those receiving a 1.5 mg daily dosage of fondaparinux. Together, these data support the treatment of low-dose fondaparinux for the prevention of VTE in Chinese patients undergoing TKA.

## 6. Limitation

Some limitations of our study were as follows: First, when it comes to diagnosing DVT in patients, venography is generally accepted as the method of choice. However, the procedure is invasive, and its repeated use is impractical (18). Since doppler ultrasonography is noninvasive and could be employed repeatedly to check for DVT, we utilized it to make the diagnosis (19). Second, patients in our research were of Chinese descent, and DVT rates may vary across ethnic groups. A study has shown similar incidences of DVT in Asians and Westerners (20). However, a low incidence of DVT has been documented in some studies in patients of Asian ethnicity undergoing joint arthroplasty (21). Therefore, we cannot consider that the incidence in Chinese differs from other populations. Third, the sample size of the randomized controlled trial was relatively small and was underpowered to identify differences in VTE incidences across distinct groups. However, our study was sufficiently supported to identify significant variations in the primary outcomes, i.e., the rate of bleeding and wound complications, and could provide important and relevant information to surgeons performing TKA in Chinese patients.

## Data Availability

The original contributions presented in the study are included in the article, further inquiries can be directed to the corresponding authors.
